# Supplementary material for: Mesolimbic dopamine adapts the rate of learning from action
Source: Nature. 2023 Jan 18;614(7947):294–302. doi: 10.1038/s41586-022-05614-z (PMC9908546; doi:10.1038/s41586-022-05614-z)
Supplement: Supplementary file 1 — Supplementary Table 1 [file 41586_2022_5614_MOESM1_ESM.pdf]

nature portfolio

https://doi.org/10.1038/s41586-022-05614-z

Supplementary information

Mesolimbic dopamine adapts the rate of learning from action

In the format provided by the authors and unedited

	trace conditioning group				cued VTA-DA stimulation	
	(Fig. 1, 2, 4, 5)			(Ext. Fig. 4)	(Fig. 3)	
mouse	control	stimLick+	stimLick-	stim. at cue	reg. stim.	large stim.
1	x				x	
2	x					
3			x			x
4	x					x
5			x		x	
6	x					x
7		x				x
8		x			x	
9	x				x	
10			x		x	
11	x					x
12		x			x	
13		x		x		x
14			x			x
15	x			x	x	
16	x			x	x	
17		x		x	x	
18			x		x	
19	x					
20			x	x	x	

Supplementary Table 1. Successive experimental group membership for each mouse. Mice #21-24 are not included above as they were only used for a single experiment, the stim+Lick+ experiments in Fig. 5g-i
