# Supplementary material for: Mesolimbic dopamine adapts the rate of learning from action
Source: Nature. 2023 Jan 18;614(7947):294–302. doi: 10.1038/s41586-022-05614-z (PMC9908546; doi:10.1038/s41586-022-05614-z)
Supplement: Supplementary file 2 — Reporting Summary [file 41586_2022_5614_MOESM2_ESM.pdf]

nature portfolio

Corresponding author(s): Joshua T. Dudman, Luke T. Coddington

Last updated by author(s): 2022/10/23

Reporting Summary

Nature Portfolio wishes to improve the reproducibility of the work that we publish. This form provides structure for consistency and transparency in reporting. For further information on Nature Portfolio policies, see our Editorial Policies and the Editorial Policy Checklist.

Statistics

For all statistical analyses, confirm that the following items are present in the figure legend, table legend, main text, or Methods section.

n/a	Confirmed	
☐	☒	The exact sample size (*n*) for each experimental group/condition, given as a discrete number and unit of measurement
☐	☒	A statement on whether measurements were taken from distinct samples or whether the same sample was measured repeatedly
☐	☒	The statistical test(s) used AND whether they are one- or two-sided<br>*Only common tests should be described solely by name; describe more complex techniques in the Methods section.*
☐	☒	A description of all covariates tested
☐	☒	A description of any assumptions or corrections, such as tests of normality and adjustment for multiple comparisons
☐	☒	A full description of the statistical parameters including central tendency (e.g. means) or other basic estimates (e.g. regression coefficient) AND variation (e.g. standard deviation) or associated estimates of uncertainty (e.g. confidence intervals)
☐	☒	For null hypothesis testing, the test statistic (e.g. *F*, *t*, *r*) with confidence intervals, effect sizes, degrees of freedom and *P* value noted<br>*Give P values as exact values whenever suitable.*
☒	☐	For Bayesian analysis, information on the choice of priors and Markov chain Monte Carlo settings
☒	☐	For hierarchical and complex designs, identification of the appropriate level for tests and full reporting of outcomes
☐	☒	Estimates of effect sizes (e.g. Cohen's *d*, Pearson's *r*), indicating how they were calculated

Our web collection on statistics for biologists contains articles on many of the points above.

Software and code

Policy information about availability of computer code

Data collection	Task-related and fiber photometry data were collected using Cerebus Central Suite (v 7.0.6.0) software from Blackrock Microsystems. Video data was collected using Fly Capture 2 software from FLIR as well as custom written python code found at www.github.com/neurojak/pySpinCapture
Data analysis	Data were analyzed and visualized using custom code in MATLAB_R2019a (requiring the Signal Processing, Image Processing, Statistics and Machine Learning toolboxes) as well as GraphPad Prism 6. The data and custom code used to generate results supporting the findings of this study are within https://github.com/DudLab - including both modeling code (https://github.com/DudLab/RNN_learnDA) and analysis code (https://github.com/DudLab/TONIC).

For manuscripts utilizing custom algorithms or software that are central to the research but not yet described in published literature, software must be made available to editors and reviewers. We strongly encourage code deposition in a community repository (e.g. GitHub). See the Nature Portfolio guidelines for submitting code & software for further information.

Data

Policy information about availability of data

All manuscripts must include a data availability statement. This statement should provide the following information, where applicable:

- Accession codes, unique identifiers, or web links for publicly available datasets
- A description of any restrictions on data availability
- For clinical datasets or third party data, please ensure that the statement adheres to our policy

The data and custom code used to generate results supporting the findings of this study are within https://github.com/DudLab - including both modeling code (https://github.com/DudLab/RNN_learnDA) and analysis code (https://github.com/DudLab/TONIC).

Human research participants

Policy information about studies involving human research participants and Sex and Gender in Research.

Reporting on sex and gender	*Use the terms sex (biological attribute) and gender (shaped by social and cultural circumstances) carefully in order to avoid confusing both terms. Indicate if findings apply to only one sex or gender; describe whether sex and gender were considered in study design whether sex and/or gender was determined based on self-reporting or assigned and methods used. Provide in the source data disaggregated sex and gender data where this information has been collected, and consent has been obtained for sharing of individual-level data; provide overall numbers in this Reporting Summary. Please state if this information has not been collected. Report sex- and gender-based analyses where performed, justify reasons for lack of sex- and gender-based analysis.*
Population characteristics	*Describe the covariate-relevant population characteristics of the human research participants (e.g. age, genotypic information, past and current diagnosis and treatment categories). If you filled out the behavioural & social sciences study design questions and have nothing to add here, write "See above."*
Recruitment	*Describe how participants were recruited. Outline any potential self-selection bias or other biases that may be present and how these are likely to impact results.*
Ethics oversight	*Identify the organization(s) that approved the study protocol.*

Note that full information on the approval of the study protocol must also be provided in the manuscript.

Field-specific reporting

Please select the one below that is the best fit for your research. If you are not sure, read the appropriate sections before making your selection.

☒ Life sciences ☐ Behavioural & social sciences ☐ Ecological, evolutionary & environmental sciences

For a reference copy of the document with all sections, see nature.com/documents/nr-reporting-summary-flat.pdf

Life sciences study design

All studies must disclose on these points even when the disclosure is negative.

Sample size	No statistical methods were used to predetermine sample size. Sample sizes were determined in accordance with existing studies measuring and manipulating dopamine activity in awake behaving animals (e.g. Eshel et al and Uchida, Nature 2015; Saunders et al and Janak, Nat. Neuro 2018, Lee et al and Masminidis Nat. Neuro 2020).
Data exclusions	A small number of mice (n=4) were removed from the study after initial data was collected, due to poor signals. Histology determined that fibers were mistargeted and/or virus expression was insufficient in each of these mice.
Replication	We did not replicate the findings here in a new group of animals, however the dataset was compiled through serial small cohorts of animals randomly assigned to experimental groups, and cohorts displayed qualitatively similar behavioral and neural learning trajectories. Behavioral and neural learning trajectories also compare well to previously published similar experiments (https://doi.org/10.1038/s41593-018-0245-7).
Randomization	Experiments were done in repeated small cohorts (n=2-4) of mice across several months. Within each cohort, mice were randomly assigned to a group (control, stimLick-, stimLick+).
Blinding	Blinding during data collection was not possible as experimenter had to rely on group identity to determine the protocol for optogenetic manipulation. Experimenters were blind to group identity during the initial stages of analysis when analysis windows were determined and custom code was established to quantify fiber photometry signals and behavioral measurements.

Reporting for specific materials, systems and methods

We require information from authors about some types of materials, experimental systems and methods used in many studies. Here, indicate whether each material, system or method listed is relevant to your study. If you are not sure if a list item applies to your research, read the appropriate section before selecting a response.

Materials & experimental systems

n/a	Involved in the study
☒	☐ Antibodies
☒	☐ Eukaryotic cell lines
☒	☐ Palaeontology and archaeology
☐	☒ Animals and other organisms
☒	☐ Clinical data
☒	☐ Dual use research of concern

Methods

n/a	Involved in the study
☒	☐ ChIP-seq
☒	☐ Flow cytometry
☒	☐ MRI-based neuroimaging

Animals and other research organisms

Policy information about studies involving animals; ARRIVE guidelines recommended for reporting animal research, and Sex and Gender in Research

Laboratory animals	We used male, adult DAT-cre x ai32 mice (12-40 weeks old) resulting from the cross of DAT-IREScre (The Jackson Laboratory stock 006660) and Ai32 (The Jackson Laboratory stock 012569) lines of mice. Mice were maintained under specific-pathogen-free conditions. Mice were housed on a free-standing, individually ventilated (~60 air changes hourly) rack (Allentown Inc, Allentown, NJ). The holding room was ventilated with 100% outside filtered air with >15 air changes hourly. Each ventilated cage (Allentown) was provided with corncob bedding (Shepard Specialty Papers, Milford, NJ), at least 8g of nesting material (Bed-r'Nest, The Andersons, Maumee, OH), and red Mouse Tunnel (Bio-Serv, Flemington, NJ). Mice were maintained on a 12:12-h (8am-8pm) light:dark cycle and recordings were done between 9am-3pm. The holding room temperature was maintained at 70±2°F with a relative humidity of 30% to 70%. Irradiated rodent laboratory chow (LabDiet 5053) was provided ad libitum. Following at least 4 days recovery from headcap implantation surgery, animals' water consumption was restricted to 1.2 mL per day for at least 3 days before training. Mice underwent daily health checks, and water restriction was eased if mice fell below 75% of their original body weight.
Wild animals	This study did not involve wild animals.
Reporting on sex	Only male animals were used for this study.
Field-collected samples	This study did not involve field-collected samples.
Ethics oversight	All procedures and animal handling were performed in strict accordance with a protocol (#19-190) approved by the Janelia Institutional Animal Care and Use Committee (IACUC, protocol 19-190) and consistent with the standards set forth by the Association for Assessment and Accreditation of Laboratory Animal Care (AALAC).

Note that full information on the approval of the study protocol must also be provided in the manuscript.
